# α-Synuclein Regulates Neuronal Cholesterol Efflux

**DOI:** 10.3390/molecules22101769

**Published:** 2017-10-19

**Authors:** Jen-Hsiang T. Hsiao, Glenda M. Halliday, Woojin Scott Kim

**Affiliations:** 1 Brain and Mind Centre, Sydney Medical School, The University of Sydney, Sydney NSW 2050, Australia; tony.hsiao@sydney.edu.au (J.-H.T.H.); glenda.halliday@sydney.edu.au (G.M.H.); 2 Neuroscience Research Australia, Sydney NSW 2031, Australia; 3 School of Medical Sciences, University of New South Wales, Sydney NSW 2052, Australia

**Keywords:** α-synuclein, cholesterol efflux, ABCA1, apolipoproteins, Parkinson’s disease

## Abstract

α-Synuclein is a neuronal protein that is at the center of focus in understanding the etiology of a group of neurodegenerative diseases called α-synucleinopathies, which includes Parkinson’s disease (PD). Despite much research, the exact physiological function of α-synuclein is still unclear. α-Synuclein has similar biophysical properties as apolipoproteins and other lipid-binding proteins and has a high affinity for cholesterol. These properties suggest a possible role for α-synuclein as a lipid acceptor mediating cholesterol efflux (the process of removing cholesterol out of cells). To test this concept, we “loaded” SK-N-SH neuronal cells with fluorescently-labelled cholesterol, applied exogenous α-synuclein, and measured the amount of cholesterol removed from the cells using a classic cholesterol efflux assay. We found that α-synuclein potently stimulated cholesterol efflux. We found that the process was dose and time dependent, and was saturable at 1.0 µg/mL of α-synuclein. It was also dependent on the transporter protein ABCA1 located on the plasma membrane. We reveal for the first time a novel role of α-synuclein that underscores its importance in neuronal cholesterol regulation, and identify novel therapeutic targets for controlling cellular cholesterol levels.

## 1. Introduction

α-Synuclein is a 140-amino acid protein produced predominantly by neurons in the brain. It is at the center of focus in understanding the etiology of a group of neurodegenerative diseases called α-synucleinopathies. These include Parkinson’s disease (PD), dementia with Lewy bodies (DLB) [1], multiple system atrophy (MSA) [2], and a number of less well-characterized neuroaxonal dystrophies [3]. The common feature of α-synucleinopathies is the presence of proteinaceous bodies containing aggregates of α-synuclein. α-Synuclein is normally present at the presynaptic terminals of neurons, where it is thought to play a role in maintaining a supply of synaptic vesicles. α-Synuclein is encoded by the *SNCA* gene and polymorphism and mutation studies of *SNCA* have provided evidence for a causal link between α-synuclein and PD [4,5,6,7]. Six missense mutations in α-synuclein have been identified in dominantly inherited PD [8,9,10,11,12]. The causal link also exists for DLB, but not for MSA [13]. Despite much research into α-synuclein, the exact physiological function of α-synuclein is still unclear.

A body of evidence indicates a possible role of α-synuclein as a lipid acceptor. There are four main characteristics of α-synuclein that point to such a role. First, α-synuclein contains 11-amino acid repeats, which contain the consensus hexamer sequence KTKEGV, which are similar to motifs present in apolipoproteins and other lipid-binding proteins [14]. Second, α-synuclein has a strong propensity to bind to lipid membranes, particularly regions enriched in cholesterol [15]. Third, α-synuclein contains two cholesterol-binding domains (residues 34–45 and 67–78), with the latter having a high affinity for cholesterol [16]. Four, α-synuclein can form structures that are similar to nascent lipoproteins [17], which are premature forms of the larger spherical lipoproteins (i.e., high density lipoprotein). Nascent lipoproteins function as lipid carriers and promote cholesterol efflux. Cholesterol efflux is a process of removing cholesterol out of cells and is controlled by the transporter protein ABCA1, located on the plasma membrane [18,19]. Typically, ABCA1 transfers cholesterol from cells onto nearby extracellular cholesterol acceptors, such as apolipoprotein A1 (apoA1) or apolipoprotein E (apoE) [20,21]. ApoA1 stimulates cholesterol efflux from peripheral cells and aberration of this process results in accumulation of cholesterol in macrophages and, consequently, atherosclerosis [18,19,22]. ApoE discs stimulate cholesterol efflux from neuronal cells [23,24]; apoE discs are nascent lipoprotein-like structures that are secreted from astrocytes in the brain [25,26,27,28].

The function of α-synuclein as a cholesterol acceptor that mediates cholesterol efflux is unknown. We therefore hypothesized that α-synuclein mediates cholesterol efflux. In this study, we loaded SK-N-SH neuronal cells with a fluorescent-labelled cholesterol and tested whether exogenously applied α-synuclein could mediate cholesterol efflux.

## 2. Results

### 2.1. Testing Whether α-Synuclein Can Mediate Cholesterol Efflux

**Figure 1:**
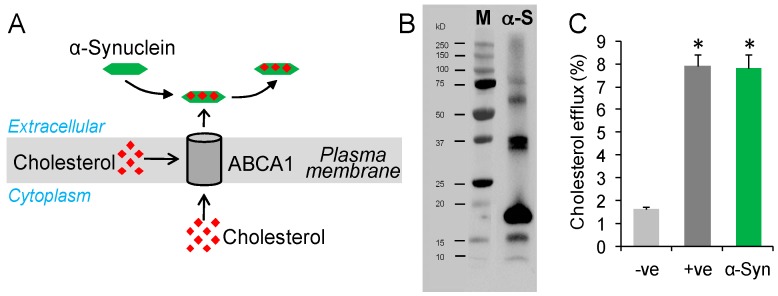
α-Synuclein mediates cholesterol efflux. (**A**) A putative model of cholesterol efflux process controlled by the transporter protein ABCA1; (**B**) A preparation of α-synuclein (α-S) protein displaying monomeric and oligomeric forms as analyzed by western blotting; standard protein marker (M); (**C**) Cholesterol efflux of SK-N-SH neuronal cells treated with exogenous α-synuclein (1.0 µg/mL) compared to −ve control (PBS) and +ve control (apoE). Data represent mean (*n* = 6) and SE as error bars, * *p* < 0.00005.

α-Synuclein has biophysical properties that are similar to lipid-binding proteins [14], and has a high affinity for cholesterol [16]. These characteristics have raised an interesting question as to whether α-synuclein could mediate cholesterol efflux (Figure 1A). Cholesterol efflux is a process of moving cholesterol out of cells onto an extracellular cholesterol acceptor, such as apoA1 and apoE. To address our question, we resuspended lyophilized α-synuclein in PBS (Figure 1B) and applied it to SK-N-SH neuronal cells that were loaded with a fluorescent-labelled cholesterol (BODIPY-cholesterol). Fluorescence was measured in media samples and in cells, and the percentage of cholesterol efflux determined. ApoE discs [23] were used as a positive control. We found that α-synuclein potently stimulated cholesterol efflux. The level of cholesterol efflux achieved by α-synuclein was similar to that of apoE discs (Figure 1C).

### 2.2. α-Synuclein Mediates Cholesterol Efflux in a Dose- and Time-Dependent Manner

**Figure 2:**
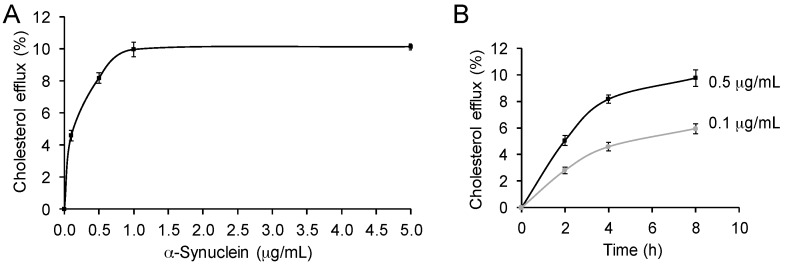
α-Synuclein mediates cholesterol efflux in a dose and time dependent manner. (**A**) Cholesterol efflux of SK-N-SH neuronal cells with increasing concentrations of α-synuclein (4 h treatment); (**B**) Cholesterol efflux of SK-N-SH neuronal cells with increasing exposure time to α-synuclein (0.1 and 0.5 µg/mL). Data represent mean (*n* = 6) and SE as error bars.

To further characterize the cholesterol efflux process mediated by α-synuclein, we assessed the effect of altering α-synuclein concentration on the process. Typically, cholesterol efflux is dependent on the concentration of the cholesterol acceptor. We once again treated SK-N-SH neuronal cells with exogenous α-synuclein and carried out cholesterol efflux assays. We found that α-synuclein stimulated cholesterol efflux in a dose-dependent manner, and the process was saturated at a concentration of 1.0 µg/mL of α-synuclein (Figure 2A). We also assessed the effect of altering the exposure time of α-synuclein on the process. Two concentrations of α-synuclein, 0.1 and 0.5 µg/mL, were used. We showed that α-synuclein stimulated cholesterol efflux in a time dependent manner (Figure 2B). These results indicate that the cholesterol efflux process, mediated by α-synuclein, is similar to that of typical cholesterol acceptors apoA1 and apoE.

### 2.3. α-Synuclein Mediates Cholesterol Efflux via ABCA1

**Figure 3:**
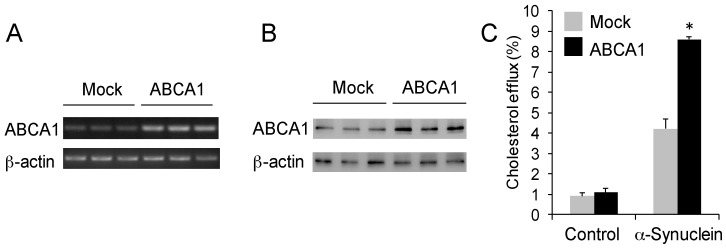
α-Synuclein mediates cholesterol efflux via ABCA1. (**A**) Agarose gel electrophoresis shows an increased expression of ABCA1 in SK-N-SH neuronal cells that were transfected with ABCA1 cDNA compared to cells transfected with empty vector (mock). The housekeeper gene β-actin was used as an internal control; (**B**) Western blotting shows an increased expression of ABCA1 in SK-N-SH neuronal cells that were transfected with ABCA1 cDNA compared to cells transfected with empty vector (mock). The housekeeper gene β-actin was used as an internal control; (**C**) Cholesterol efflux of SK-N-SH neuronal cells transfected with either ABCA1 or mock cDNA and treated with α-synuclein or PBS (−ve control) for 4 h. Data represent mean (*n* = 6) and SE as error bars, * *p* < 0.0005.

Cholesterol efflux is an active process that is controlled by the transporter protein ABCA1 located on the plasma membrane. ABCA1 is a member of ATP-binding cassette subfamily A (ABCA) that specializes in transporting lipids across membranes [29]. ApoA1- or apoE-mediated cholesterol efflux is dependent on ABCA1 [20,21,23]. To test if α-synuclein-mediated cholesterol efflux is also dependent on ABCA1, we transfected SK-N-SH neuronal cells with ABCA1 cDNA to increase ABCA1 expression, and then treated the cells with exogenous α-synuclein. Firstly, we confirmed the increased expression of ABCA1 in the transfected cells (Figure 3A,B). We showed that the level of cholesterol efflux was significantly increased in the ABCA1-overexpressing cells compared to mock-transfected cells (Figure 3C). The increase was 104%, which correlates strongly with the higher ABCA1 expression. There was no significant change in cholesterol efflux in the absence of exogenous α-synuclein (control) (Figure 3C), indicating that ABCA1 alone does not induce cholesterol efflux.

## 3. Discussion

Cholesterol homeostasis in the brain is increasingly recognized as being important for normal brain function. Cholesterol is an integral component of membranes, contributing to membrane structure and function. It is also a precursor to metabolites that are involved in multiple metabolic pathways. The level of cholesterol in cells is tightly regulated, as high levels are toxic. The level is controlled at the point of endogenous synthesis or at the point of exogenous uptake. A third mechanism of control is an active removal of cholesterol out of cells, which is known as cholesterol efflux. ApoA1 and apoE are cholesterol acceptors that play an integral part in the cholesterol efflux process. Aberration in neuronal cholesterol transport has been implicated in a number of neurodegenerative processes. For example, decreases in neuronal cholesterol efflux resulted in increases in the production of the neurotoxic amyloid-β peptides [23]. The role of α-synuclein as a cholesterol acceptor is unknown. Whether neuronal cholesterol efflux is altered in α-synucleinopathies is also unknown.

The lipid-binding characteristics of α-synuclein suggest a possible role of α-synuclein as a cholesterol acceptor that mediates cholesterol efflux. In this study, we tested the capability of α-synuclein to mediate cholesterol efflux. We applied exogenous α-synuclein to SK-N-SH neuronal cells and assessed whether it could remove cholesterol using a classic cholesterol efflux assay. We showed, for the first time, that α-synuclein potently stimulated cholesterol efflux. Aspects of the cholesterol efflux process mediated by α-synuclein were similar to those of the typical cholesterol acceptors apoA1 and apoE. Firstly, the level of cholesterol efflux achieved by α-synuclein was similar to that of other acceptors [23,30]. Secondly, the cholesterol efflux process was dose dependent and saturable at high concentrations of α-synuclein. Thirdly, the process was time dependent, i.e., the longer the exposure the higher the efflux. Fourthly, the process was dependent on ABCA1, the transmembrane protein that controls the transfer of cholesterol out of cells. These data indicate that α-synuclein mediates cholesterol efflux via ABCA1.

A close physical interaction between ABCA1 and cholesterol acceptor is believed to be a prerequisite for cholesterol efflux. Topological studies, based on cross-linking assays, have demonstrated that the distance between ABCA1 and apoA1 is less than 7 angstroms, which suggests a direct protein-protein interaction [30,31]. An alternative model of interaction that has been proposed is that apoA1 does not directly interact with ABCA1 but rather binds to the plasma membrane, and thereby alters the physiochemical property of the membrane which facilitates cholesterol efflux via ABCA1 [32]. Virtually nothing is known about the interaction between α-synuclein and ABCA1. In vitro studies have shown that α-synuclein binds to regions of membranes that are enriched in cholesterol [15], and that the 11-amino acid repeats, containing the consensus hexamer motif, play a pivotal role in the binding process [33]. Research is needed to determine the nature of interaction between α-synuclein and the plasma membrane, and whether a direct interaction between α-synuclein and ABCA1 is a prerequisite for cholesterol efflux.

In our cholesterol efflux assay, we utilized a fluorescent-labelled cholesterol called BODIPY-cholesterol, which is dipyrromethene boron difluoride linked to the carbon 24 of cholesterol [34]. In the past, a radioactive-labelled cholesterol (^3^H-cholesterol) was commonly used in cholesterol efflux assays. When the two were compared side-by-side in a recent cholesterol efflux study, BODIPY-cholesterol produced generally higher levels of efflux, although all other aspects of cholesterol efflux process were similar [35]. In our study BODIPY-cholesterol also produced higher levels of efflux compared to our previous studies with ^3^H-cholesterol in the same cells (SK-N-SH) with the same cholesterol acceptor (apoE discs) [23]. It is likely that the photophysical property of BODIPY allows higher emission of light/radiation [34,36], which could be related to the higher efflux readings. BODIPY-cholesterol has been shown to have similar characteristics as untagged cholesterol [37], and has been used extensively in cholesterol trafficking studies [38]. BODIPY-cholesterol is non-radioactive, and therefore it is readily applicable to high throughput studies.

In conclusion, we have tested whether α-synuclein could mediate cholesterol efflux and found that it potently stimulates cholesterol efflux. This process was dependent on the transmembrane transporter ABCA1. We reveal for the first time a novel role of α-synuclein and provide new insights into the potential pathomechanism of α-synucleinopathies.

## 4. Materials and Methods

### 4.1. Cell Culture

SK-N-SH neuronal cells were obtained from the ATCC (Manassas, VA, USA) and were cultured in Dulbecco’s modified Eagle’s medium (DMEM) containing 10% fetal calf serum, 1% Glutamax, 0.5% glucose, 100 IU/mL penicillin and 100 μg/mL streptomycin at 37 °C in humidified air containing 5% CO_2_. Cell culture media and additives were obtained from Invitrogen (Melbourne, Australia), unless stated otherwise.

### 4.2. Cholesterol Efflux Assay

Cholesterol efflux assay was carried out as previously described [35]. Briefly, SK-N-SH neuronal cells were cultured in 48-well plates as described above. The cells were then labeled with BODIPY-cholesterol (Avanti Polar Lipids, Alabaster) for 1 h, washed with phosphate-buffered saline (PBS) three times, and equilibrated with DMEM containing 0.1% (*w*/*v*) bovine serum albumin (BSA) for 2 h. The cells were rinsed once more in PBS, and then incubated in serum-free media with or without exogenous α-synuclein (1.0 µg/mL) (AnaSpec, Fermont, CA, USA), which was prepared from lyophilized powder in sterile PBS and kept at 4 °C. ApoE discs (15 µg/mL) were used as a positive control [23]. The fluorescence of media and cell lysate samples was measured using a Molecular Device M2 plate reader (excitation 482 nm, emission 515 nm) and the percentage cholesterol efflux calculated.

### 4.3. Western Blotting

α-Synuclein preparations were separated on SDS-PAGE gels and transferred onto 0.45 μm nitrocellulose membranes at 100 volts for 30 min. Membranes were blocked with PBS containing 5% non-fat dry milk and probed with α-synuclein antibody (1:1000, mouse monoclonal, BD biosciences, North Ryde, Australia) overnight at 4 °C. They were then washed three times in PBS containing 0.1% Tween 20 and incubated with horseradish peroxidase-conjugated secondary antibody (1:2000, Dako, Carpinteria, CA, USA) for 2 h. Signals were detected using enhanced chemiluminescence and X-ray films (ECL, GE Healthcare, Buckinghamshire, UK).

### 4.4. Transfection

Transient transfection was performed using Lipofectamine 2000 and Opti-MEM I (Invitrogen, Melbourne, Australia) following the manufacturer’s protocol. Briefly, cells were seeded at 90% confluence in 48-well plates using antibiotic-free media and transfected with ABCA1 cDNA or empty vector (control). They were incubated for 24 h prior to cholesterol efflux assay.

### 4.5. RNA Extraction and Quantitative PCR

RNA was isolated using TRIzol reagent (Invitrogen) following the manufacturer’s protocol. All procedures were carried out using RNase-free reagents and consumables. One microgram of RNA was reverse transcribed into cDNA using Moloney-murine leukemia virus (M-MLV) reverse transcriptase and random primers (Promega, Madison, WI, USA) in 20 μL reaction volume. Quantitative PCR (qPCR) assays were carried out using a Mastercycler ep realplex S (Eppendorf, Sydney, Australia) and the fluorescent dye SYBR Green (Bio-Rad, 1725270, Sydney, Australia), following the manufacturer’s protocol. Briefly, each reaction (20 μL) contained 1× mastermix, 5 pmoles of primers and 1 μL of cDNA template. Amplification was carried out with 40 cycles of 94 °C for 15 s and 60 °C for 1 min. Gene expression was normalized to the housekeeper gene β-actin. A no-template control was included for each PCR amplification assay. The level of expression for each gene was calculated using the comparative threshold cycle (Ct) value method using the formula 2^−ΔΔ*C*t^ (where ΔΔ*C*_t_ = Δ*C*_t_ sample−Δ*C*_t_ reference). PCR products were visualized using 1% agarose gel electrophoresis.

### 4.6. Statistical Analysis

Data presented are expressed as mean ± SE shown by the error bars. Statistical significance was determined using Student’s *t*-test with *p* values < 0.05 considered significant.

## References

[1] Spillantini M.G., Schmidt M.L., Lee V.M., Trojanowski J.Q., Jakes R., Goedert M. (1997). Alpha-synuclein in lewy bodies. Nature.

[2] Gai W.P., Power J.H., Blumbergs P.C., Blessing W.W. (1998). Multiple-system atrophy: A new alpha-synuclein disease?. Lancet.

[3] Newell K.L., Boyer P., Gomez-Tortosa E., Hobbs W., Hedley-Whyte E.T., Vonsattel J.P., Hyman B.T. (1999). Alpha-synuclein immunoreactivity is present in axonal swellings in neuroaxonal dystrophy and acute traumatic brain injury. J. Neuropathol. Exp. Neurol..

[4] Pals P., Lincoln S., Manning J., Heckman M., Skipper L., Hulihan M., Van den Broeck M., De Pooter T., Cras P., Crook J. (2004). Alpha-synuclein promoter confers susceptibility to parkinson’s disease. Ann. Neurol..

[5] Rajput A., Vilarino-Guell C., Rajput M.L., Ross O.A., Soto-Ortolaza A.I., Lincoln S.J., Cobb S.A., Heckman M.G., Farrer M.J. (2009). Alpha-synuclein polymorphisms are associated with parkinson’s disease in a saskatchewan population. Mov. Disord. Off. J. Mov. Disord. Soc..

[6] Pankratz N., Wilk J.B., Latourelle J.C., DeStefano A.L., Halter C., Pugh E.W., Doheny K.F., Gusella J.F., Nichols W.C., Foroud T. (2009). Genomewide association study for susceptibility genes contributing to familial parkinson disease. Hum. Genet..

[7] Maraganore D.M., de Andrade M., Elbaz A., Farrer M.J., Ioannidis J.P., Kruger R., Rocca W.A., Schneider N.K., Lesnick T.G., Lincoln S.J. (2006). Collaborative analysis of alpha-synuclein gene promoter variability and parkinson disease. J. Am. Med. Assoc..

[8] Polymeropoulos M.H., Lavedan C., Leroy E., Ide S.E., Dehejia A., Dutra A., Pike B., Root H., Rubenstein J., Boyer R. (1997). Mutation in the alpha-synuclein gene identified in families with parkinson’s disease. Science.

[9] Kruger R., Kuhn W., Muller T., Woitalla D., Graeber M., Kosel S., Przuntek H., Epplen J.T., Schols L., Riess O. (1998). Ala30pro mutation in the gene encoding alpha-synuclein in parkinson’s disease. Nat. Genet..

[10] Zarranz J.J., Alegre J., Gomez-Esteban J.C., Lezcano E., Ros R., Ampuero I., Vidal L., Hoenicka J., Rodriguez O., Atares B. (2004). The new mutation, e46k, of alpha-synuclein causes Parkinson and lewy body dementia. Ann. Neurol..

[11] Proukakis C., Dudzik C.G., Brier T., MacKay D.S., Cooper J.M., Millhauser G.L., Houlden H., Schapira A.H. (2013). A novel alpha-synuclein missense mutation in Parkinson disease. Neurology.

[12] Lesage S., Anheim M., Letournel F., Bousset L., Honore A., Rozas N., Pieri L., Madiona K., Durr A., Melki R. (2013). G51d alpha-synuclein mutation causes a novel parkinsonian-pyramidal syndrome. Ann. Neurol..

[13] Sailer A., Scholz S.W., Nalls M.A., Schulte C., Federoff M., Price T.R., Lees A., Ross O.A., Dickson D.W., Mok K. (2016). A genome-wide association study in multiple system atrophy. Neurology.

[14] Clayton D.F., George J.M. (1998). The synucleins: A family of proteins involved in synaptic function, plasticity, neurodegeneration and disease. Trends Neurosci..

[15] Fortin D.L., Troyer M.D., Nakamura K., Kubo S., Anthony M.D., Edwards R.H. (2004). Lipid rafts mediate the synaptic localization of alpha-synuclein. J. Neurosci. Off. J. Soc. Neurosci..

[16] Fantini J., Carlus D., Yahi N. (2011). The fusogenic tilted peptide (67–78) of alpha-synuclein is a cholesterol binding domain. Biochim. Biophys. Acta.

[17] Varkey J., Mizuno N., Hegde B.G., Cheng N., Steven A.C., Langen R. (2013). Alpha-synuclein oligomers with broken helical conformation form lipoprotein nanoparticles. J. Biol. Chem..

[18] Brooks-Wilson A., Marcil M., Clee S.M., Zhang L.H., Roomp K., van Dam M., Yu L., Brewer C., Collins J.A., Molhuizen H.O. (1999). Mutations in abc1 in tangier disease and familial high-density lipoprotein deficiency. Nat. Genet..

[19] Bodzioch M., Orso E., Klucken J., Langmann T., Bottcher A., Diederich W., Drobnik W., Barlage S., Buchler C., Porsch-Ozcurumez M. (1999). The gene encoding atp-binding cassette transporter 1 is mutated in tangier disease. Nat. Genet..

[20] Oram J.F., Lawn R.M., Garvin M.R., Wade D.P. (2000). Abca1 is the camp-inducible apolipoprotein receptor that mediates cholesterol secretion from macrophages. J. Biol. Chem..

[21] Wang N., Silver D.L., Costet P., Tall A.R. (2000). Specific binding of apoa-i, enhanced cholesterol efflux, and altered plasma membrane morphology in cells expressing abc1. J. Biol. Chem..

[22] McNeish J., Aiello R.J., Guyot D., Turi T., Gabel C., Aldinger C., Hoppe K.L., Roach M.L., Royer L.J., de Wet J. (2000). High density lipoprotein deficiency and foam cell accumulation in mice with targeted disruption of atp-binding cassette transporter-1. Proc. Natl. Acad. Sci. USA.

[23] Kim W.S., Rahmanto A.S., Kamili A., Rye K.A., Guillemin G.J., Gelissen I.C., Jessup W., Hill A.F., Garner B. (2007). Role of abcg1 and abca1 in regulation of neuronal cholesterol efflux to apolipoprotein e discs and suppression of amyloid-beta peptide generation. J. Biol. Chem..

[24] Fu Y., Hsiao J.H., Paxinos G., Halliday G.M., Kim W.S. (2015). Abca5 regulates amyloid-beta peptide production and is associated with Alzheimer’s disease neuropathology. J. Alzheimers Dis..

[25] Pitas R.E., Boyles J.K., Lee S.H., Hui D., Weisgraber K.H. (1987). Lipoproteins and their receptors in the central nervous system. Characterization of the lipoproteins in cerebrospinal fluid and identification of apolipoprotein b,e(ldl) receptors in the brain. J. Biol. Chem..

[26] Fagan A.M., Holtzman D.M., Munson G., Mathur T., Schneider D., Chang L.K., Getz G.S., Reardon C.A., Lukens J., Shah J.A. (1999). Unique lipoproteins secreted by primary astrocytes from wild type, apoe (−/−), and human apoe transgenic mice. J. Biol. Chem..

[27] LaDu M.J., Gilligan S.M., Lukens J.R., Cabana V.G., Reardon C.A., Van Eldik L.J., Holtzman D.M. (1998). Nascent astrocyte particles differ from lipoproteins in csf. J. Neurochem..

[28] Ladu M.J., Reardon C., Van Eldik L., Fagan A.M., Bu G., Holtzman D., Getz G.S. (2000). Lipoproteins in the central nervous system. Ann. N. Y. Acad. Sci..

[29] Kim W.S., Weickert C.S., Garner B. (2008). Role of atp-binding cassette transporters in brain lipid transport and neurological disease. J. Neurochem..

[30] Fitzgerald M.L., Morris A.L., Rhee J.S., Andersson L.P., Mendez A.J., Freeman M.W. (2002). Naturally occurring mutations in the largest extracellular loops of abca1 can disrupt its direct interaction with apolipoprotein a-i. J. Biol. Chem..

[31] Fitzgerald M.L., Mendez A.J., Moore K.J., Andersson L.P., Panjeton H.A., Freeman M.W. (2001). Atp-binding cassette transporter a1 contains an nh2-terminal signal anchor sequence that translocates the protein’s first hydrophilic domain to the exoplasmic space. J. Biol. Chem..

[32] Chambenoit O., Hamon Y., Marguet D., Rigneault H., Rosseneu M., Chimini G. (2001). Specific docking of apolipoprotein a-i at the cell surface requires a functional abca1 transporter. J. Biol. Chem..

[33] Segrest J.P., Jones M.K., De Loof H., Brouillette C.G., Venkatachalapathi Y.V., Anantharamaiah G.M. (1992). The amphipathic helix in the exchangeable apolipoproteins: A review of secondary structure and function. J. Lipid Res..

[34] Li Z., Mintzer E., Bittman R. (2006). First synthesis of free cholesterol-bodipy conjugates. J. Org. Chem..

[35] Sankaranarayanan S., Kellner-Weibel G., de la Llera-Moya M., Phillips M.C., Asztalos B.F., Bittman R., Rothblat G.H. (2011). A sensitive assay for abca1-mediated cholesterol efflux using bodipy-cholesterol. J. Lipid Res..

[36] Li Z., Bittman R. (2007). Synthesis and spectral properties of cholesterol- and fty720-containing boron dipyrromethene dyes. J. Org. Chem..

[37] Klose C., Ejsing C.S., Garcia-Saez A.J., Kaiser H.J., Sampaio J.L., Surma M.A., Shevchenko A., Schwille P., Simons K. (2010). Yeast lipids can phase-separate into micrometer-scale membrane domains. J. Biol. Chem..

[38] Holtta-Vuori M., Uronen R.L., Repakova J., Salonen E., Vattulainen I., Panula P., Li Z., Bittman R., Ikonen E. (2008). Bodipy-cholesterol: A new tool to visualize sterol trafficking in living cells and organisms. Traffic.

